# Selective Hematological Profiles in Drug-Naïve Early Autism: Clinical and Developmental Correlates

**DOI:** 10.3390/biomedicines14061237

**Published:** 2026-05-29

**Authors:** Dilek Altun Varmış, Cumali Yüksekkaya, Hülya Binokay, Serkan Güneş, Elif Gözde Yüce Antepüzümü, Yunus Kıllı, Nazmiye İnce, Hamide Kübra Özlük

**Affiliations:** 1 Department of Child and Adolescent Psychiatry, Adana City Training and Research Hospital, University of Health Sciences, 01010 Adana, Turkey; 2 Department of Biostatistics, Faculty of Medicine, Cukurova University, 01330 Adana, Turkey

**Keywords:** autism spectrum disorder, childhood, hemogram, systemic immune inflammation, iron metabolism, Denver II, Childhood Autism Rating Scale, sex differences, basophil

## Abstract

**Background/Objectives**: Peripheral biomarkers for autism spectrum disorder (ASD) have shown mixed results in previous studies. In this study, complete blood count-derived immune-inflammatory markers, iron and micronutrient levels, and thyroid function were compared between drug-naïve preschoolers newly diagnosed with ASD and healthy controls. Additionally, the relationships between these markers, symptom severity, and developmental skills were examined. **Methods**: This retrospective case–control study included 62 children with ASD (aged 24–72 months) and 61 age-matched healthy controls. Symptom severity, behavioral traits, and developmental status were assessed using the Childhood Autism Rating Scale (CARS), Autism Behavior Checklist (ABC), and Denver II Developmental Screening Test (DDST), respectively. Composite inflammatory indices were calculated from hemogram data. Statistical analyses incorporated Holm–Bonferroni corrections for multiple comparisons and sex-stratified exploratory analyses of conditional associations using 95% bootstrap confidence intervals based on 5000 resamples. **Results**: Children with ASD demonstrated significantly lower mean corpuscular volume (MCV; d = 0.66, adj. *p* = 0.019), lower mean platelet volume (MPV; d = 0.58, adj. *p* = 0.034), and higher absolute lymphocyte counts (LYMPH; d = 1.10, adj. *p* = 0.019). Initial group differences in ferritin, serum iron, and transferrin saturation did not survive adjustment (adj. *p* > 0.05). Composite inflammatory indices were not significantly associated with clinical or developmental scores. Higher CARS and ABC scores correlated with lower personal–social and language scores on the DDST (*p* < 0.01). Furthermore, exploratory sex-stratified, conditional association analyses suggested preliminary basophil- and lymphocyte-related patterns in girls; however, these findings are strictly hypothesis-generating due to the small female sample size (n = 12). **Conclusions**: Newly diagnosed, drug-naïve preschoolers with ASD showed a distinct baseline blood profile, including lower MCV and MPV and higher lymphocyte counts. Clinical challenges were most evident in personal–social and language domains. While the primary diagnostic value of routine hemograms in this context appears limited, the exploratory sex-stratified basophil- and lymphocyte-related patterns require validation in adequately powered future cohorts.

## 1. Introduction

Autism spectrum disorder (ASD) is a diverse and complex neurodevelopmental disorder that begins in early childhood. It is characterized by persistent social communication difficulties and restricted and repetitive patterns of behavior, interests, or activities [1]. The clinical presentation, developmental trajectory, and associated biological features vary substantially between individuals [2]. This heterogeneity suggests that a single explanatory mechanism does not account for ASD and supports a multi-pathway model [2].

Accessible peripheral biomarkers have attracted increasing attention in ASD research because they are inexpensive, minimally invasive, and potentially useful in clinical settings [3]. However, findings regarding erythrocyte indices, iron parameters, and routine hematological inflammatory markers remain inconsistent [3]. Some studies have reported higher rates of iron deficiency and lower ferritin levels in children with ASD [4,5,6]. Similarly, erythrocyte-related parameters have been associated with symptom severity or adaptive functioning in some studies; however, their standalone diagnostic or prognostic values remain limited [2,6].

A similar uncertainty applies to composite inflammatory indices derived from complete blood count (CBC) [7]. Although these hemogram-based markers may reflect peripheral immune variations across neurodevelopmental conditions, their specificity to ASD remains unclear [8]. Current evidence suggests that immune-related signals may be concentrated in biologically or clinically defined subgroups rather than generalized across all ASD cases [9,10,11,12]. Accordingly, peripheral hematological variations in ASD may be conditional, developmentally sensitive, and shaped by the sample composition [9,10,11,12].

From a developmental psychopathology perspective, we adopt a neuroimmune-threshold framework. This framework posits that peripheral immune markers do not directly index central nervous system inflammation but rather reflect systemic physiological states that may interact with neurodevelopmental processes during sensitive periods [13,14]. Within this framework, we conceptualize the peripheral hematological profile primarily as a risk-context indicator. Rather than serving as a diagnostic tool with direct clinical utility, it reflects a systemic physiological background that co-occurs with, and may indirectly shape, specific neurodevelopmental trajectories during the critical preschool window. Guided by this framework, the present study distinguishes between (a) acute systemic inflammation, (b) chronic low-grade immune dysregulation reflected in composite cellular indices, and (c) selective hematological variation involving specific cell populations, such as lymphocytes or platelets [13,14]. The preschool period is a critical window during which social reciprocity, communication, and self-regulatory capacities are rapidly reorganized. Consequently, developmental timing is essential for interpreting the significance of peripheral biomarkers [13,14,15].

Although some recent studies have linked immune markers to symptom-related measures [11,12], evidence connecting routine hematological indices to the developmental burden of preschool ASD remains limited [15,16]. This issue is clinically important because preschool ASD often shows uneven neurodevelopmental profiles, with a particularly marked burden in the language and personal–social domains, and specific language milestones may be informative for severity stratification [15,16]. Therefore, peripheral blood parameters should be interpreted cautiously, not as direct indicators of central neuroinflammation, but as low-cost correlates, the meaning of which may depend on developmental timing, subgroup features, and sex [11,12,15,16].

Sex may be particularly relevant in this context [17,18]. The marked male predominance in ASD has long raised the possibility that some biological pathways differ by sex [17,18]. Recent transcriptomic findings support this view by identifying distinct molecular signatures in children with ASD [17,18]. Emerging serum immune-marker studies also suggest cytokine-related immune variation in ASD [19]. However, most ASD biomarker studies have focused on monocyte subsets [11], cytokines [19], and proteomic panels [12], whereas basophils have received comparatively little attention, and their role in ASD remains unclear [11,12,19]. However, there is substantial epidemiological evidence linking ASD with a higher prevalence of atopic and allergic conditions. For instance, large-scale studies have demonstrated that children with ASD are significantly more likely to experience food, respiratory, and skin allergies compared to typically developing children [20]. Furthermore, recent findings confirm a strong association between ASD and atopic comorbidities, such as asthma and eczema [21]. Given that basophils are key effector cells in allergic responses and type 2 inflammation, the increased burden of allergic comorbidities in ASD provides a compelling physiological rationale to investigate basophil variations in this population.

In our study, we compared blood counts (hematological parameters) and micronutrient levels between drug-naïve preschoolers (aged 24–72 months) newly diagnosed with ASD and healthy controls. We also examined whether these markers were associated with clinician-rated severity, parent-reported behavioral traits, and developmental performance across the personal–social, fine-motor-adaptive, language, and gross-motor domains.

Therefore, the overall goal of this study was to evaluate comprehensive hematological parameters, composite inflammatory indices, and micronutrient levels in newly diagnosed, drug-naïve preschoolers with ASD compared to healthy controls. By examining how these peripheral markers correlate with clinician-rated symptom severity, parent-reported behavioral traits, and standardized developmental functioning, we aimed to clarify the clinical relevance of routine blood indices in early ASD. Additionally, we explored whether selected hematological variables were involved in exploratory conditional association patterns between behavioral burden and clinical severity within a sex-stratified framework.

The novelty of the present study lies in three unique elements. First, it strictly focuses on a newly diagnosed, entirely drug-naïve preschool cohort, thereby isolating baseline hematological profiles from the confounding effects of psychotropic medications. Second, it maps these biological parameters not only to global autism severity but also to specific, continuously measured neurodevelopmental domains (e.g., language and personal–social skills). Finally, this is among the first studies to explore basophil-related conditional association patterns within a sex-stratified framework in early ASD.

## 2. Materials and Methods

### 2.1. Study Design

We conducted a single-center, retrospective case–control study at the Child and Adolescent Psychiatry and Pediatrics Departments of Adana City Training and Research Hospital. Data were retrieved from the Hospital Information Management System (HIMS) and archived patient files. The study was reported in accordance with the Strengthening the Reporting of Observational Studies in Epidemiology (STROBE) checklist (see Supplementary Materials, Table S1). Because this was a retrospective chart-review study, no study-specific clinical assessments, laboratory testing, or blood collection was performed. Instead, the study used routine blood tests requested at the first clinical presentation to aid in differential diagnosis, evaluate current metabolic status, and establish a baseline before any potential pharmacological intervention.

### 2.2. Participants

We included children aged 24–72 months who were evaluated between 1 September 2022 and 1 January 2026. The ASD group consisted of children diagnosed with ASD according to DSM-5 criteria by a child and adolescent psychiatrist. Drug-naïve status was confirmed through parent report and HIMS pharmacy records, with no prior or current use of psychotropics, stimulants, or antiepileptics. In our clinic, the Childhood Autism Rating Scale (CARS), the Autism Behavior Checklist (ABC), and the Denver II Developmental Screening Test are routinely used to evaluate preschool children with suspected ASD. The control group consisted of children of similar age attending pediatric well-child visits, with no known psychiatric, neurodevelopmental, or neurological disorder, and Denver II results within normal limits.

Children in both groups were excluded if they had acute infection or fever at the time of evaluation, chronic inflammatory, autoimmune, hematological, or malignant disease, severe systemic illness, immunosuppressive treatment, or insufficient clinical or laboratory records. Additional phenotypic subgrouping was not performed because the available sample size, particularly for girls, would have produced sparse cells and unstable estimates; subgroup analysis was therefore limited to exploratory sex-stratified models. The final sample comprised 62 children with ASD (50 boys and 12 girls) and 61 healthy controls (33 boys and 28 girls). Residual sporadic missing laboratory values were below 2% per parameter and were handled with pairwise deletion.

Because the study was retrospective and the sample size was modest, these analyses were treated as exploratory rather than causal. Although ASD is heterogeneous, we did not divide the cohort into multiple phenotypic subgroups because further stratification would have produced small cell sizes and unstable estimates. Instead, we reduced heterogeneity by focusing on newly diagnosed, drug-naïve preschoolers and limited subgroup analysis to exploratory sex-stratified models.

### 2.3. Clinical Assessments and Scales

Given the retrospective design of the study, no new assessments were performed. Instead, the CARS, ABC, and Denver II evaluations that were most closely aligned with the diagnostic assessment were retrieved from the clinical records and used in the analyses.

#### 2.3.1. Childhood Autism Rating Scale (CARS)

The severity of autism spectrum disorder (ASD) symptoms was determined using the total score from the CARS, a 15-item clinician-rated observational instrument used to identify children with autism and evaluate symptom severity [22]. This assessment relies on a combination of direct clinical observation and caregiver-provided information. The scale evaluates 15 specific domains, including relating to people, imitation, emotional response, body use, and verbal/non-verbal communication. Each item is scored on a Likert scale ranging from 1 (normal for the child’s age) to 4 (severely abnormal). The total score ranges from 15 to 60, with higher scores indicating greater autism severity; a cutoff of 30 is typically used for clinical identification. The Turkish adaptation of this scale has demonstrated satisfactory validity and reliability [23].

#### 2.3.2. Autism Behavior Checklist (ABC)

Behavioral characteristics were evaluated using the ABC score. The ABC is a 57-item parent- or caregiver-reported screening tool specifically designed to evaluate the frequency and severity of autistic behavioral traits [24]. The items are distributed across five symptom domains: sensory, relating, body and object use, language, and social/self-help skills. Each item is scored using a weighted system (ranging from 1 to 4) based on its predictive association with ASD. The sum of these weighted scores yields a total behavioral problem score, where higher values reflect a higher frequency of atypical behaviors. Psychometric data confirming the validity and reliability of the Turkish version of this checklist are available in the literature [25].

#### 2.3.3. Denver II Developmental Screening Test

Developmental status was evaluated using the Denver II Developmental Screening Test (DDST), a standardized instrument consisting of 125 items that assesses child development from birth to 6 years of age [26,27]. It is administered through a structured combination of direct clinical observation and structured caregiver reports. The test evaluates four independent domains: personal–social, fine motor-adaptive, language, and gross motor skills. Rather than employing categorical pass/fail or normal/abnormal interpretations of the DDST, the developmental age equivalents (expressed in months) for each domain were analyzed as continuous variables in the present study. This quantitative approach allowed for a more granular assessment of the developmental burden across specific domains. However, it should be noted that while using continuous variables increases statistical power and granularity, it may complicate the direct translation of these findings into the categorical ‘delay’ or ‘no delay’ classifications typically used in routine clinical practice.

### 2.4. Laboratory Assessments

Routine laboratory results obtained closest to the clinical evaluation were systematically retrieved from the Hospital Information Management System (HIMS). Hematological parameters included hemoglobin, mean corpuscular volume (MCV), platelet count (PLT), absolute neutrophil (NEUT), lymphocyte (LYMPH), monocyte (MONO), eosinophil (EOS), and basophil (BASO) counts, mean platelet volume (MPV), and red cell distribution width (RDW). Biochemical parameters included C-reactive protein (CRP), ferritin, serum iron, total iron-binding capacity (TIBC), transferrin saturation (TS), vitamin B12, folate, thyroid-stimulating hormone (TSH), and free thyroxine (fT4). All laboratory analyses had been performed in the same accredited hospital laboratory using standardized automated methods as part of routine clinical care. Hormone and vitamin parameters were measured using the Roche Cobas 8000 system (Roche Diagnostics, Mannheim, Germany). Complete blood count (CBC) was analyzed using the MINDRAY 5200 hematology analyzer (Shenzhen, China). The laboratory panel was limited to routinely available tests already present in the records. No additional blood samples were obtained for research purposes. Consequently, non-routine biomarkers such as catecholamines, brain-derived neurotrophic factor (BDNF), interleukin-6 (IL-6), and tumor necrosis factor-alpha (TNF-α) were not measured.

### 2.5. Hemogram-Derived Inflammation Indices

To further explore the peripheral immune-inflammatory balance, several key indices were calculated from hemogram data. All indices were computed using absolute cell counts (×10^3^/µL) to ensure consistency. These included the neutrophil-to-lymphocyte ratio (NLR), platelet-to-lymphocyte ratio (PLR), and monocyte-to-lymphocyte ratio (MLR). More complex composite indices were also derived: the systemic immune-inflammation index (SII), calculated as (neutrophil × platelet)/lymphocyte; systemic inflammation response index (SIRI), determined by (neutrophil × monocyte)/lymphocyte; and aggregate index of systemic inflammation (AISI), computed as (neutrophil × platelet × monocyte)/lymphocyte [7]. These indices offer a more holistic framework for understanding systemic inflammation [3,7].

### 2.6. Statistical Analysis

Statistical analyses were performed using SPSS version 26.0 (IBM Corp., Armonk, NY, USA). The distribution of the data was examined using the Kolmogorov–Smirnov test. For comparisons of continuous variables, the independent-samples *t*-test was used when the assumptions for parametric testing were satisfied; otherwise, the Mann–Whitney U test was applied. Categorical variables were compared using the chi-square test or Fisher’s exact test, where appropriate. Cohen’s d was reported as a measure of effect size for continuous group differences.

Correlations between biological markers, clinical scale scores, and Denver developmental domains were assessed using Pearson or Spearman correlations, depending on the variables’ distributional characteristics. To account for multiple comparisons in the primary group analyses, the Holm–Bonferroni step-down procedure was applied to adjust *p*-values across the family of all primary hematological and comparisons between the ASD and control groups. The exploratory correlation and conditional association analyses were not adjusted for multiple comparisons and should therefore be interpreted strictly as hypothesis-generating. To address the significant difference in sex distribution between the groups, an Analysis of Covariance (ANCOVA) was subsequently conducted for variables that survived the Holm–Bonferroni correction. In these models, the clinical diagnosis (ASD vs. Control) was entered as the fixed factor, and sex was entered as a categorical adjustment factor, allowing us to estimate the sex-adjusted main effect of the diagnosis.

Exploratory conditional association analyses were conducted. In these models, the total ABC score was entered as the predictor and the total CARS score as the outcome. It is important to note that both behavioral traits (ABC) and clinical severity (CARS) were assessed concurrently during the initial diagnostic evaluation. Therefore, the designation of ABC as a predictor and CARS as an outcome in these path analyses reflects an exploratory structural convention to test conditional association patterns, rather than implying any temporal or causal precedence. LYMPH and BASO counts were tested separately as intermediary variables, and sex was included as a moderator of the path between the intermediary variable and the outcome. Thus, the association between ABC scores and the intermediary variables was estimated for the overall ASD group. These analyses were cross-sectional and exploratory; therefore, they should not be interpreted as demonstrating causal mediation or temporal precedence. These conditional process analyses were conducted using the PROCESS macro for SPSS (v4.x, Model 14), which specifically estimates models where the moderator acts on the path between the intermediary variable and the outcome. Conditional association estimates were evaluated using 95% bootstrap confidence intervals based on 5000 resamples. This high number of resamples is the recommended methodological standard to ensure the stability and robustness of the confidence intervals for conditional association estimates [28]. A two-sided *p*-value < 0.05 was considered significant.

In these exploratory models, sex was included as a moderator only for the path between the intermediary variable and the outcome (path b), while the path from the predictor to the intermediary variable (path a) was estimated in the pooled sample. This constrained modeling choice was a necessary analytical compromise to preserve model stability given the critically small female subgroup (n = 12). However, because the pooled sample is predominantly male, the shared path a is heavily driven by male data. Consequently, the female-specific conditional association estimates are not derived from fully independent female-specific parameters and must be interpreted with extreme caution.

### 2.7. Ethics Statement

This study was approved by the Scientific Research Ethics Committee of Adana City Training and Research Hospital (protocol code: 1071; approval date: 19 February 2026). Owing to the retrospective chart review design, the Ethics Committee waived informed consent. No blood sample was collected solely for research purposes; all laboratory data were derived from previously obtained clinical records. All patient data were strictly anonymized and de-identified prior to analysis to ensure confidentiality.

## 3. Results

### 3.1. Demographic and Clinical Characteristics

A total of 123 children were included in the study: 61 controls (49.6%) and 62 children with ASD (50.4%). Mean age was 49.70 ± 14.66 months (ASD: 49.87 ± 15.44; controls: 49.52 ± 13.96; *p* = 0.896). Sex distribution differed significantly between groups; the proportion of males was higher in the ASD group (80.6% vs. 54.1%; *p* = 0.002).

### 3.2. Hematological and Biochemical Comparisons

**Figure 1:**
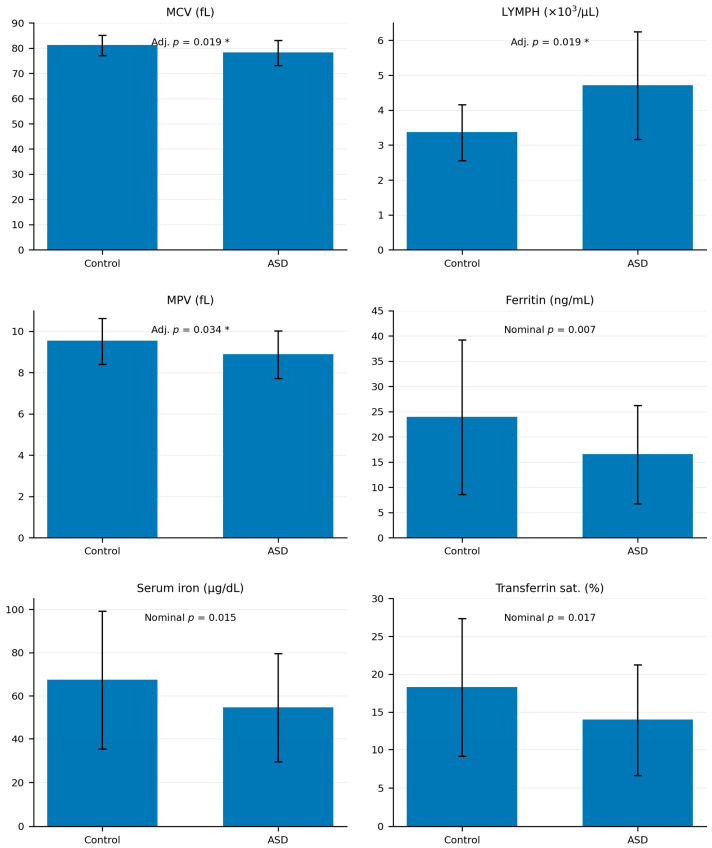
Mean ± SD group comparisons for key hematological and iron-related variables differentiating ASD and control groups. MCV, LYMPH, and MPV retained significance after Holm–Bonferroni correction, whereas ferritin, serum iron, and transferrin saturation were nominally significant only. * *p* < 0.05.

The results are presented in Table 1 according to the three tiers. (a) Confirmed findings (surviving Holm–Bonferroni correction): MCV (d = 0.66), LYMPH (d = 1.10), and MPV (d = 0.58) differed significantly and retained significance after correction. (b) Uncorrected trends: Ferritin (d = 0.58; *p* = 0.007; adj. *p* = 0.112), serum iron (d = 0.45; *p* = 0.015; adj. *p* = 0.225), and transferrin saturation (d = 0.52; *p* = 0.017; adj. *p* = 0.238) were numerically lower in the ASD group but did not withstand correction and should not be interpreted as evidence of iron deficiency. (c) Null findings: All remaining parameters (hemoglobin, PLT, NEUT, MONO, EOS, BASO, RDW, TSH, fT4, folate, CRP, vitamin B12, and TIBC) showed no significant differences (all *p* > 0.05). While Table 1 presents the primary unadjusted group comparisons, the marked sex imbalance between the groups necessitated a covariate-adjusted approach to confirm the baseline findings. To ensure that the observed differences were not driven by the unequal sex distribution between the groups, a sex-adjusted ANCOVA was performed on the three parameters that survived multiple-comparison correction. After adjusting for sex, the main effect of ASD diagnosis remained statistically significant for MCV (F(1, 120) = 16.425, adjusted *p* < 0.001), MPV (F(1, 120) = 8.233, adjusted *p* = 0.005), and absolute lymphocyte count (LYMPH) (F(1, 120) = 34.275, adjusted *p* < 0.001). These adjusted estimates indicate that the distinct hematological profile observed in the ASD group is independent of the sample’s sex composition. Additionally, exploratory correlation analyses confirmed that age in months did not significantly correlate with MCV, MPV, or LYMPH within our sample (*p* > 0.05). Furthermore, introducing age as an additional continuous covariate into the ANCOVA models did not alter the direction, magnitude, or statistical significance of the diagnostic main effects, confirming the robustness of the profile across the 24–72 months window. A visual summary of the key group comparisons is presented in Figure 1.

### 3.3. Correlations Between Hematological Indices, Clinical Severity, and Developmental Domains

**Figure 2:**
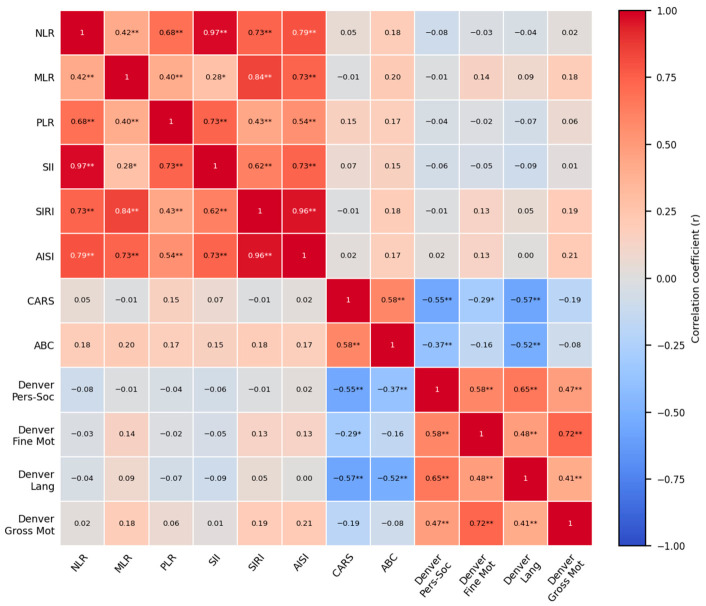
Correlation heatmap showing associations among composite inflammatory indices, clinical scale scores, and Denver II developmental domains in the ASD group. (* *p* < 0.05; ** *p* < 0.01).

These relationships are visualized in Figure 2. The composite inflammatory indices (NLR, MLR, PLR, SII, SIRI, and AISI) were strongly positively intercorrelated (|r| range: 0.28–0.97). None of these indices were significantly associated with CARS, ABC, or any Denver developmental domain (all |r| ≤ 0.21, all *p* > 0.05). CARS and ABC scores showed a moderate positive intercorrelation (r = 0.58, *p* < 0.01). CARS negatively correlated with Denver personal–social (r = −0.55, *p* < 0.01), fine motor (r = −0.29, *p* < 0.05), and language (r = −0.57, *p* < 0.01) domains; ABC negatively correlated with Denver personal–social (r = −0.37, *p* < 0.01) and language (r = −0.52, *p* < 0.01). The full correlation matrix is presented in Table 2.

### 3.4. Exploratory Sex-Stratified Conditional Association Patterns

Exploratory sex-stratified conditional association analyses are provided in the Supplementary Materials (Tables S2 and S3). These preliminary models explored LYMPH and BASO counts as intermediary variables between behavioral traits (ABC) and clinical severity (CARS). While the models suggested potential conditional association patterns in the female subgroup, these findings must be interpreted with extreme caution. The female ASD subgroup (n = 12) was severely underpowered, resulting in notably wide confidence intervals for the estimated conditional association patterns. The large coefficients observed (particularly for basophils) likely reflect measurement instability across a very narrow physiological range and limited sample size rather than a definitive biological signal. Therefore, these sex-stratified patterns are reported strictly as hypothesis-generating observations for future research.

**Table 2:** Correlations between systemic immune-inflammation indices, clinical scales, and Denver II developmental domains in the patient group.

Variable	NLR	MLR	PLR	SII	SIRI	AISI	CARS Total	ABC Total	Denver Pers-Soc (mo)	Denver Fine Mot (mo)	Denver Lang (mo)	Denver Gross Mot (mo)
NLR	1											
MLR	0.42 **	1										
PLR	0.68 **	0.40 **	1									
SII	0.97 **	0.28 *	0.73 **	1								
SIRI	0.73 **	0.84 **	0.43 **	0.62 **	1							
AISI	0.79 **	0.73 **	0.54 **	0.73 **	0.96 **	1						
CARS total	0.05	−0.01	0.15	0.07	−0.01	0.02	1					
ABC total	0.18	0.20	0.17	0.15	0.18	0.17	0.58 **	1				
Denver pers-soc (mo)	−0.08	−0.01	−0.04	−0.06	−0.01	0.02	−0.55 **	−0.37 **	1			
Denver fine mot (mo)	−0.03	0.14	−0.02	−0.05	0.13	0.13	−0.29 *	−0.16	0.58 **	1		
Denver lang (mo)	−0.04	0.09	−0.07	−0.09	0.05	0.00	−0.57 **	−0.52 **	0.65 **	0.48 **	1	
Denver gross mot (mo)	0.02	0.18	0.06	0.01	0.19	0.21	−0.19	−0.08	0.47 **	0.72 **	0.41 **	1

**Note.** Correlation coefficients: * *p* < 0.05. ** *p* < 0.01. ABC, Autism Behavior Checklist; AISI, aggregate index of systemic inflammation; CARS, Childhood Autism Rating Scale; MLR, monocyte-to-lymphocyte ratio; NLR, neutrophil-to-lymphocyte ratio; PLR, platelet-to-lymphocyte ratio; SII, systemic immune-inflammation index; SIRI, systemic inflammation response index.

## 4. Discussion

Overall, these findings indicate a selective hematological pattern rather than a broad inflammatory signature. Simultaneously, the variables most strongly associated with symptom burden came from developmental domains, particularly language and personal–social functioning.

### 4.1. Iron Metabolism and Erythrocyte Indices

The finding of a lower MCV in the absence of overt anemia suggests subtle erythrocyte-level variation rather than a clear iron deficiency [2,29]. Previous studies have reported a higher prevalence of iron deficiency, along with lower ferritin, hemoglobin, hematocrit, and MCV-related parameters in individuals with ASD [4,5,6]. Notably, Gunes et al. found lower hemoglobin, hematocrit, iron, and MCV levels in children with ASD than in healthy controls, with the greatest reductions observed in preschool-aged children [6]. However, in our sample, the iron-related pattern did not remain significant after correction for multiple comparisons. This suggests that iron-related differences may reflect context-dependent variations rather than a stable deficiency profile [30,31]. These findings are consistent with the broader literature indicating that peripheral iron markers are sensitive to sample composition and methodological differences [30,31]. Adams et al. likewise reported that routine vitamin and mineral measures may remain within normal ranges even when more detailed metabolic markers show abnormalities [32].

Furthermore, it is clinically important to clarify that the present study was not designed to formally diagnose iron deficiency. Our retrospective methodology did not include systematic assessments of dietary intake, nor did it allow for inflammation-adjusted interpretations of ferritin, which are critical for an accurate clinical diagnosis. Therefore, the absence of Holm–Bonferroni-corrected significance for ferritin, serum iron, and transferrin saturation should be viewed strictly within these methodological constraints. Future prospective studies aiming to rigorously test iron deficiency hypotheses in early ASD should incorporate comprehensive nutritional assessments, dietary diaries, and more advanced, inflammation-adjusted iron panels.

### 4.2. Lymphocyte Elevation and Selective Peripheral Immune Variation

Elevated lymphocyte counts in the ASD group likely reflect selective immune cell redistribution rather than generalized acute systemic inflammation [33,34]. Importantly, although the group-level effect size for this difference was substantial (d = 1.10), the mean absolute lymphocyte counts in the ASD cohort (4.70 × 10^3^/µL) remained well within the standard pediatric reference ranges for the preschool age group. Therefore, this variation should not be interpreted as a definitive immune ‘signature’ and its clinical meaning or diagnostic utility at the individual patient level is currently limited. This view aligns with comparable CRP levels across groups and the lack of meaningful associations between inflammatory indices and clinical severity or developmental domains.

Overall, rather than indicating a homogeneous inflammatory phenotype, our results point to a more selective pattern of peripheral immune variation. It is crucial to clarify that the term ‘selective’ is not used here to imply a unique or ASD-specific biological signature. Rather, it explicitly refers to our observation that alterations were restricted to certain cell populations (i.e., lymphocytes and platelets) in the absence of group-level differences in acute-phase reactants (CRP) or composite inflammatory indices. This localized variation is highly compatible with the concept that peripheral immune changes in ASD are largely subgroup-dependent or context-dependent, rather than a universal diagnostic feature [33,34]. Meta-analytic evidence similarly suggests that lymphocyte subsets and monocyte/macrophage-related pathways may be altered in ASD, although not consistently across all cases [33,34].

From a mechanistic perspective, this pattern fits the neuroimmune threshold framework, which proposes that peripheral immune differentiation and neurodevelopment interact indirectly without implying that routine blood findings directly reflect inflammation in the brain [13,14]. It is important to emphasize that we did not measure cytokine levels or markers of microglial activation in the present study. Therefore, peripheral cytokine signaling and microglial pathways are presented here strictly as illustrative background models drawn from the literature, rather than processes directly inferred from our dataset. Within this theoretical background, animal models demonstrate that cytokines such as IL-6, TNF-α, and IFN-γ can cross the blood–brain barrier or activate microglial cells via vagal afferents, offering a plausible conceptual framework for understanding how selective peripheral immune variations might coincide with neurodevelopmental burden [13,14].

### 4.3. Composite Inflammatory Indices and Heterogeneity

The absence of associations between composite inflammatory indices and clinical severity in our sample suggests that these measures reflect a broader peripheral hematological background rather than an ASD-specific clinical burden [3,8]. This differs from the subgroup-based findings reported by Ellul et al., who observed NLR abnormalities in ASD cases with maternal immune activation [9]. Together, these results support a model in which inflammatory signals in ASD are subgroup-dependent rather than generalizable across all early diagnosed and drug-naïve cases [9]. The developmental stage may be one reason for this variability. Elevated NLR has been reported in younger individuals with ASD [35], whereas adolescent cohorts have not consistently shown a similar inflammatory profile [34]. This pattern suggests that hematological markers may shift across developmental periods.

Dietary factors may also contribute to this heterogeneity. Recent evidence suggests that a higher dietary inflammatory burden may accompany ASD and correlate with selected composite inflammatory measures [36]. Because dietary patterns were not systematically assessed in our sample, some of the observed biological variability may reflect unmeasured nutritional influences. Accordingly, the null associations observed here should not be interpreted as evidence against immune involvement in ASD in general. Instead, a crucial distinction should be made: while these composite indices did not correlate with functional burden or symptom severity in our overarching, unstratified cohort, they may still be informative within other clinically defined subgroups, such as those characterized by a history of maternal immune activation, specific metabolic comorbidities, or distinct neuroinflammatory phenotypes. Therefore, rather than negating the role of immune mechanisms, our findings suggest that these specific peripheral indices are not reliable universal markers of clinical burden across all early ASD presentations.

### 4.4. MPV and Platelet-Related Signaling

Lower MPV was another consistent finding; however, its interpretation requires caution [37]. Previous studies on MPV in ASD have produced mixed results, with some reporting lower MPV and others finding no significant group differences [35,38]. MPV is a context-sensitive marker associated with platelet activation, thrombosis, and inflammation [37]. In our sample, a reduced MPV may represent a platelet-related signal rather than a disorder-specific marker.

This interpretation is biologically plausible because platelets are the main peripheral reservoir of serotonin [39] and play an important role in thrombosis and inflammatory regulation [37,40]. Given the longstanding literature on serotonergic abnormalities in ASD [39], altered MPV may capture one aspect of broader platelet-related physiological variation. At the same time, MPV alterations have also been reported in other psychiatric conditions [41], so this finding should not be overinterpreted as specific to ASD. Furthermore, MPV is known to be influenced by allergic diseases, atopy, and low-grade subclinical infections, which are frequently comorbid in preschool children. Because we did not systematically assess the atopic or allergic status of the participants beyond the strict exclusion of overt acute infections and severe chronic inflammatory diseases, this unmeasured variability further precludes any direct causal interpretations linking MPV exclusively to ASD neurobiology [37,40,42,43,44]. Overall, the present findings support viewing MPV as one component of a broader hematological pattern rather than as a standalone biomarker [35,37,38,39,40,41,42,43,44].

While it is conventional to discuss erythrocyte indices, platelet parameters, and immune cells separately, their concurrent alteration in our cohort—specifically lower MCV, lower MPV, and higher lymphocyte counts—suggests a shared underlying mechanistic pathway. This distinct triad may reflect a systemic physiological environment driven by chronic, low-grade oxidative stress and immune dysregulation. Previous literature extensively documents elevated oxidative stress in ASD [45], which can disrupt lipid membrane integrity and turnover, potentially manifesting as reduced cellular volumes in both erythrocytes (MCV) and platelets (MPV). Concurrently, this systemic low-grade inflammatory state may stimulate selective cellular redistribution, explaining the elevated lymphocyte counts. Furthermore, because platelets are the primary peripheral reservoir for serotonin [39], the reduced MPV observed here might directly intersect with the well-established hypothesis of disrupted serotonergic signaling in ASD. While early studies, such as Gunes et al. [6], noted isolated reductions in MCV, and meta-analyses by Arteaga-Henríquez et al. [33] highlighted subset-specific lymphocyte elevations, our findings emphasize the necessity of viewing these parameters collectively. Together, they outline a peripheral blood profile shaped by the continuous interplay between oxidative membrane dynamics, serotonergic regulation, and systemic immune thresholds during early neurodevelopment [13,14,39,45].

### 4.5. Vitamin and Thyroid Parameters

The lack of significant group differences in vitamin B12, folate, TSH, and fT4 further supports the impression of a selective rather than a generalized biological profile. In this cohort, these parameters did not meaningfully distinguish children with ASD from the controls. This is important because it helps prevent mechanistic overinterpretation. Micronutrient factors, such as vitamin B12 and folate, may still be relevant in certain ASD subgroups [46,47]. Thyroid-related mechanisms may also be important under specific developmental conditions [48,49]. However, our data do not support mechanistic conclusions based on thyroid or vitamin status. A more cautious interpretation is that these markers were not prominent differentiators in this study sample.

### 4.6. Clinical Severity and Developmental Functioning

The correlation pattern observed in this study suggests that developmental domains provide a clearer account of clinical burden than peripheral biomarkers. Integrating these findings with our neuroimmune-threshold framework, we view the distinct hematological profile observed in the ASD group (lower MCV/MPV and higher lymphocytes) neither as a direct correlate of specific neurodevelopmental trajectories nor as a generalized physiological burden. Instead, we interpret it mainly as a risk-context indicator—a reflection of a broader systemic physiological background [13,14]. Because these peripheral markers do not linearly track with clinical severity, their direct clinical utility for individual patient stratification remains limited at this stage. The strong negative correlations between CARS and the Denver personal–social (r = −0.55) and language (r = −0.57) domains indicate that greater symptom severity is closely linked to impairments in core developmental functions. This is consistent with large preschool ASD datasets showing uneven neurodevelopmental profiles and the strongest relationships between core symptoms and the language and personal–social domains [15]. Our findings add to the literature by showing that, within this sample, the internal correlation structure of developmental domains offered a stronger clinical signal than hematological variation. In early ASD, developmental organization appears to account for the clinical burden more consistently than peripheral biology.

ABC scores were inversely associated with personal–social and language performance, whereas CARS scores were related to fine motor functioning. Together, these findings suggest that an increasing symptom burden is accompanied most consistently by poorer functioning in domains central to early ASD presentation [15,16]. From a clinical perspective, the most useful message of this study may lie less in the biological differences than in the developmental domains that most reliably organize impairment.

Accordingly, routine hematological parameters should not be interpreted as stand-alone indicators of autism severity in newly diagnosed preschool-aged children. Developmental assessment, particularly in the personal–social and language domains, appears to offer a more dependable index of current clinical burden. The present findings also do not support obtaining blood samples solely to estimate autism severity in routine preschool assessments.

### 4.7. Exploratory Sex-Stratified Conditional Association Patterns

Exploratory analyses suggested preliminary sex-stratified conditional association patterns involving lymphocyte and basophil counts. These patterns were suggested primarily in the female subgroup; however, given the very small female ASD subgroup, they should not be interpreted as evidence of stable sex-specific biological effects or clinically meaningful sex differences. Rather, they suggest that sex may be relevant for future hypothesis-driven studies of peripheral hematological variation and clinical burden in ASD, consistent with broader evidence that sex may shape molecular features of ASD [17,18].

To our knowledge, this is among the first studies to explore basophil-related conditional association patterns in ASD within a sex-stratified framework. Previous biomarker studies in ASD have focused mainly on monocyte subsets [11], cytokines [19], and multi-protein panels [12], whereas basophils have received relatively little attention [11,12,19]. Given the documented overlap between ASD and allergic or atopic comorbidities discussed above [20,21], basophil-related variation remains a biologically plausible area for future investigation. Basophils can release histamine and type 2 cytokines, including IL-4 and IL-13 [50,51,52]; in the present context, these mediators should be viewed as a biologically plausible background for future neuroimmune studies rather than as direct mechanistic evidence. Histamine also functions as a central neuromodulator involved in arousal, attention, and wakefulness [53,54].

However, these findings require considerable caution. The female ASD subgroup included only 12 participants, which is too small to support stable subgroup estimates of these conditional associations. Therefore, sex-stratified findings should be regarded as hypothesis-generating rather than confirmatory, are not suitable for clinical inference at this stage, and require validation in adequately powered, sex-balanced prospective cohorts.

### 4.8. Limitations

This study had several limitations. First, its retrospective and cross-sectional design precluded temporal or causal inference. Second, although the primary group comparisons were unadjusted, sex-adjusted and age-checked sensitivity analyses supported the robustness of the findings. Nevertheless, residual confounding from the marked sex imbalance cannot be fully excluded. Third, subgroup analyses, particularly those involving girls with ASD, were severely underpowered. Simulation studies suggest that detecting moderate mediation-type or conditional process effects with 80% statistical power (α = 0.05) typically requires a minimum of 70 to 100 participants per group [55]. With only 12 girls in our ASD cohort, these sex-stratified findings must be viewed strictly as preliminary and hypothesis-generating. Future studies must prioritize large, sex-balanced cohorts that meet these power thresholds to confirm whether these peripheral hematological association patterns are reproducible across sex strata.

Fourth, developmental functioning was assessed using domain-based Denver II indices derived from a screening instrument rather than a comprehensive developmental test. Fifth, potentially important covariates, including dietary patterns, supplement use, gastrointestinal symptoms, atopic/allergic status, subclinical inflammatory conditions, socioeconomic context, and psychiatric comorbidities, were not systematically assessed. Specifically, the lack of anthropometric (e.g., body mass index [BMI], adiposity) and lifestyle data (e.g., physical activity levels) limits our ability to fully disentangle the observed hematological variations from broader nutritional or metabolic influences. Because the study relied on existing clinically obtained laboratory data to avoid additional burden in this vulnerable age group, non-routine biomarkers such as catecholamines, BDNF, IL-6, and TNF-α were unavailable. Similarly, non-routine nutritional and malabsorption markers, such as zinc, copper, and celiac screening, were also unavailable.

Sixth, because data were retrieved retrospectively from routine clinical records, we could not systematically verify the exact pre-analytical conditions of the blood draws, such as strict adherence to fasting protocols. This unmeasured pre-analytical variability could potentially influence the levels of diet-sensitive metabolic parameters, such as vitamin B12, folate, and serum iron, and might partially account for the lack of significant group differences in these specific markers.

Furthermore, our strict exclusion criteria which omitted children with acute infections, chronic autoimmune or inflammatory diseases, and those on immunosuppressive treatments were necessary to isolate baseline hematological profiles. However, because altered immunological responses and inflammatory comorbidities are prevalent within the broader ASD population, excluding these individuals limits the generalizability of our findings. Future longitudinal studies should specifically incorporate and stratify ASD cohorts with these co-occurring immunological conditions to better understand how altered immune functions contribute to the clinical and developmental heterogeneity of ASD.

Finally, detailed immune measures such as cytokine panels, lymphocyte subsets, and basophil activation markers were not available. Taken together, these constraints mean that the present findings should be understood as a focused peripheral laboratory profile rather than a comprehensive neuroimmune characterization of ASD.

## 5. Conclusions

In this retrospective case–control study, newly diagnosed, drug-naïve preschool children with ASD demonstrated a selective peripheral hematological profile characterized by significantly lower MCV and MPV alongside higher absolute lymphocyte counts compared to healthy controls. This profile was highly selective, as no generalized alterations were observed in CRP, thyroid function, vitamin B12, folate, or composite inflammatory indices. Crucially, clinical symptom severity and behavioral traits were more strongly and consistently associated with continuous developmental domains, specifically language and personal–social functioning, than with any peripheral blood parameters. These findings suggest that routine hemogram indices function as low-cost, context-dependent group correlates reflecting a systemic physiological background rather than serving as standalone diagnostic tools for individual clinical stratification.

Building on the present findings, future research should move toward a more integrated and longitudinal framework to clarify the complex neuroimmune landscape of ASD. First, longitudinal studies are essential to determine whether the selective hematological variations observed here are stable markers or developmentally transient states. Second, prioritizing larger, sex-balanced cohorts is critical to reach the statistical power required to confirm the exploratory conditional association patterns involving basophils and lymphocytes identified in this exploratory analysis. We explicitly note that these exploratory sex-stratified conditional association patterns are not suitable for clinical inference at this stage; rather, they are offered strictly as a hypothesis-generating basis for designing adequately powered, sex-stratified prospective studies. Third, future studies should transition from routine hematological indices to the direct measurement of specific immune mediators, such as cytokine panels, lymphocyte subsets, and basophil activation markers, to provide a deeper mechanistic characterization. Additionally, a more detailed characterization of environmental factors—including dietary patterns, gastrointestinal comorbidities, and socioeconomic context—will be vital for isolating the biological signatures of ASD from unmeasured nutritional and metabolic influences. Finally, rather than excluding children with co-occurring allergic or inflammatory conditions, future research should explicitly include and stratify these subgroups to understand how altered immune functions contribute to the clinical and developmental heterogeneity of the disorder. By combining peripheral biological measures with comprehensive developmental assessments beyond screening tools, future research may clarify whether these signals have reproducible clinical relevance in well-characterized ASD subgroups.

## Figures and Tables

**Table 1:** Comparison of systemic immune-inflammation and micronutrient/thyroid measurements between the patient and control groups.

Measurement	Control (n = 61)	Patient (n = 62)	*p*	Adj. *p*	Cohen’s d
Hemoglobin (g/dL)	12.10 ± 1.68	12.09 ± 0.96	0.999	>0.999	<0.01
MCV (fL)	81 ± 4	78 ± 5	<0.001	0.019 *	0.66
PLT (×10^3^/µL)	347 ± 53	354 ± 88	0.600	>0.999	0.10
NEUT (×10^3^/µL)	3.28 ± 0.93	4.10 ± 2.26	0.160	>0.999	0.48
LYMPH (×10^3^/µL)	3.35 ± 0.80	4.70 ± 1.54	<0.001	0.019 *	1.10
MONO (×10^3^/µL)	0.68 ± 0.23	0.75 ± 0.28	0.136	>0.999	0.28
EOS (×10^3^/µL)	0.23 ± 0.15	0.32 ± 0.30	0.182	>0.999	0.38
BASO (×10^3^/µL)	0.05 ± 0.03	0.06 ± 0.04	0.834	>0.999	0.03
MPV (fL)	9.51 ± 1.11	8.86 ± 1.15	0.002	0.034 *	0.58
RDW (%)	38.65 ± 2.47	38.78 ± 2.58	0.765	>0.999	0.05
TSH (µIU/mL)	2.387 ± 1.097	2.035 ± 0.974	0.070	0.910	0.34
fT4 (ng/dL)	0.930 ± 0.131	0.943 ± 0.146	0.594	>0.999	0.10
Ferritin (ng/mL)	23.85 ± 15.32	16.44 ± 9.77	0.007	0.112	0.58
Folate (ng/mL)	13.34 ± 4.12	13.26 ± 5.41	0.924	>0.999	0.02
CRP (mg/L)	2.15 ± 0.89	2.22 ± 1.12	0.689	>0.999	0.07
Vitamin B12 (pg/mL)	327 ± 129	340 ± 151	0.618	>0.999	0.09
Serum iron (µg/dL)	67.18 ± 31.93	54.40 ± 25.07	0.015	0.225	0.45
TIBC (µg/dL)	314.90 ± 49.64	321.47 ± 68.70	0.545	>0.999	0.11
Transferrin sat. (%)	18.22 ± 9.11	13.91 ± 7.30	0.017	0.238	0.52

**Note.** Data are presented as the mean ± SD. Adj. *p* = Holm–Bonferroni corrected *p*-value. * Retained significance after correction. Cohen’s d = standardized mean difference. BASO, basophil; CRP, C-reactive protein; EOS, eosinophil; fT4, free thyroxine; LYMPH, absolute lymphocyte count; MCV, mean corpuscular volume; MONO, monocyte; MPV, mean platelet volume; NEUT, neutrophil; PLT, platelet count; RDW, red cell distribution width; TIBC, total iron-binding capacity; TSH, thyroid-stimulating hormone.

## Data Availability

The data supporting the findings of this study are available from the corresponding author upon reasonable request, owing to privacy restrictions.

## Supplementary Materials

The following supporting information can be downloaded at: https://www.mdpi.com/article/10.3390/biomedicines14061237/s1, Table S1. STROBE Checklist for Observational Studies; Table S2. Exploratory conditional association patterns involving LYMPH in the ABC–CARS relationship stratified by sex; Table S3. Exploratory conditional association patterns involving BASO in the ABC–CARS relationship stratified by sex.
