# Supplementary material for: Selective Hematological Profiles in Drug-Naïve Early Autism: Clinical and Developmental Correlates
Source: Biomedicines. 2026 May 29;14(6):1237. doi: 10.3390/biomedicines14061237 (PMC13296381; doi:10.3390/biomedicines14061237)
Supplement: Supplementary file 1 [file biomedicines-14-01237-s001.zip › biomedicines-4249521-supplementary.pdf]

biomedicines

MDPI

Table S1. STROBE Checklist for Observational Studies (Case-Control Study)

Completed checklist for the manuscript "Selective Hematological Profiles in Drug-Naïve Early Autism: Clinical and Developmental Correlates".

Item No.	Recommendation	Location in Manuscript
1a	Indicate the study design in the title or abstract	Title; Abstract (p. 1)
1b	Provide an informative and balanced abstract summary	Abstract (p. 1)
2	Explain the scientific background and rationale	Introduction (pp. 3–5)
3	State specific objectives and any prespecified hypotheses	Introduction, final paragraphs (p. 5)
4	Present key elements of study design early in the paper	Methods 2.1 (p. 5)
5	Describe setting, locations, and relevant dates	Methods 2.2 (pp. 5–6)
6a	Describe eligibility criteria and the sources/methods of case ascertainment and control selection	Methods 2.2 (pp. 5–6)
6b	For matched studies, give matching criteria and number of controls per case	Not applicable (study not matched)
7	Clearly define outcomes, exposures, predictors, potential confounders, and effect modifiers	Methods 2.3–2.6 (pp. 6–11)
8	For each variable of interest, give data sources and measurement methods	Methods 2.3–2.5 (pp. 6–9)
9	Describe efforts to address potential sources of bias	Methods 2.2; Discussion 4.8 (pp. 5–6, 24–25)
10	Explain how study size was arrived at	Methods 2.2 (pp. 5–6)
11	Explain how quantitative variables were handled in the analyses	Methods 2.3, 2.6 (pp. 6–11)
12a	Describe all statistical methods, including those used to control for confounding	Methods 2.6 (pp. 9–11)
12b	Describe methods used to examine subgroups and interactions	Methods 2.6; Results 3.4; Supplementary Tables S2–S3 (pp. 9–11, 13; Supplementary Materials)
12c	Explain how missing data were addressed	Methods 2.2 (pp. 5–6)
12d	If applicable, explain how matching of cases and controls was addressed	Not applicable (study not matched)
12e	Describe any sensitivity analyses	Sex-adjusted ANCOVA sensitivity analyses were performed for primary variables surviving multiple-comparison correction, and age-marker correlations were evaluated (see Section 2.6 and Section 3.2; pp. 9–12).
13a	Report numbers of individuals at each stage of the study	Results 3.1 (pp. 11–12)
13b	Give reasons for non-participation at each stage	Not applicable in retrospective chart review
13c	Consider use of a flow diagram	Not used
14a	Give characteristics of study participants and information on exposures/confounders	Results 3.1–3.2; Table 1 (pp. 11–14)
14b	Indicate number of participants with missing data for each variable	Methods 2.2 (pp. 5–6); Results/Table 1 (pp. 13–14)
15	Report numbers in each exposure category or summary measures of exposure	Results 3.1–3.4; Tables 1–2 (pp. 11–18); Supplementary Tables S2–S3
16a	Give unadjusted and, if applicable, adjusted estimates and their precision	Results 3.2–3.4; Tables 1–2 (pp. 12–18); Supplementary Tables S2–S3

 https://doi.org/10.3390/biomedicines14061237

Item No.	Recommendation	Location in Manuscript
16b	Report category boundaries when continuous variables were categorized	Not applicable
16c	If relevant, translate estimates into absolute risk for a meaningful period	Not applicable
17	Report other analyses done (subgroups, interactions, sensitivity analyses)	Results 3.2–3.4; Table 2 (pp. 12–18); Supplementary Tables S2–S3
18	Summarise key results with reference to study objectives	Discussion (pp. 18–25)
19	Discuss limitations, including potential bias or imprecision	Discussion 4.8 (pp. 24–25)
20	Give a cautious overall interpretation of results	Discussion; Conclusions (pp.18–26)
21	Discuss the generalisability of the results	Discussion 4.8; Conclusions (pp.24–26)
22	Give the source of funding and the role of the funders	Funding statement (p.27)

Note: Page numbers refer to the current proofread manuscript layout and the present placement of the display items; the final published layout may differ slightly.

https://doi.org/10.3390/biomedicines14061237

Table S2. Exploratory conditional association patterns involving LYMPH in the ABC–CARS relationship stratified by sex.

Sex	Effect Type	Path	B	SE	Lower 95% CI	Upper 95% CI	p
Female	Conditional	ABC → LYMPH → CARS	−0.040	0.019	−0.077	−0.004	–
	Component	ABC → LYMPH	−0.021	0.008	−0.037	−0.005	0.011
	Component	LYMPH → CARS	1.929	0.467	1.014	2.843	<0.001
	Direct	ABC → CARS	0.208	0.081	0.050	0.367	0.010
	Total	ABC → CARS	0.152	0.083	−0.010	0.314	0.066
Male	Conditional	ABC → LYMPH → CARS	−0.005	0.010	−0.025	0.015	–
	Component	ABC → LYMPH	−0.021	0.008	−0.037	−0.005	0.011
	Component	LYMPH → CARS	0.236	0.467	−0.679	1.150	0.614
	Direct	ABC → CARS	0.181	0.034	0.114	0.248	<0.001
	Total	ABC → CARS	0.176	0.034	0.110	0.243	<0.001

Note. B = unstandardized coefficient; SE = standard error; CI = 95% confidence interval derived from 5,000 bootstrap resamples. These analyses were strictly exploratory and cross-sectional; no causal ordering was implied. Statistical significance for the conditional association estimates is established when the bootstrapped 95% CI does not contain zero; therefore, p-values are not reported for these specific estimates. To preserve model stability given the substantially underpowered female ASD subgroup (n = 12), the predictor-to-mediator component (ABC → LYMPH) was estimated in the pooled sample. The findings in girls should be interpreted strictly as hypothesis-generating. ABC, Autism Behavior Checklist; CARS, Childhood Autism Rating Scale; LYMPH, absolute lymphocyte count.

https://doi.org/10.3390/biomedicines14061237

Table S3. Exploratory conditional association patterns involving BASO in the ABC–CARS relationship stratified by sex.

Sex	Effect Type	Path	B †	SE	Lower 95% CI	Upper 95% CI	p
Female	Conditional	ABC → BASO → CARS	−0.060	0.026	−0.112	−0.008	–
	Component	ABC → BASO	−0.0012	0.0004	−0.0019	−0.0004	0.017
	Component	BASO → CARS	108.694	15.752	77.821	139.566	<0.001
	Direct	ABC → CARS	0.207	0.076	0.058	0.356	0.006
	Total	ABC → CARS	0.152	0.083	−0.010	0.314	0.066
Male	Conditional	ABC → BASO → CARS	−0.019	0.012	−0.041	0.004	–
	Component	ABC → BASO	−0.0012	0.0004	−0.0019	−0.0004	0.017
	Component	BASO → CARS	33.817	15.752	2.944	64.689	0.032
	Direct	ABC → CARS	0.195	0.032	0.133	0.258	<0.001
	Total	ABC → CARS	0.176	0.034	0.110	0.243	<0.001

Note. B = unstandardized coefficient; SE = standard error; CI = 95% confidence interval based on 5,000 bootstrap resamples. † BASO coefficients appear numerically large because the basophil counts were measured on a very narrow scale (0.00–0.20 $\times 10^3/\mu L$). For each 0.01 $\times 10^3/\mu L$ increase in BASO, the model estimated an increase of approximately 1.09 CARS points in females and 0.34 points in males. Statistical significance for the conditional association estimates is established when the bootstrapped 95% CI does not contain zero; therefore, p-values are not reported for these specific estimates. To preserve model stability given the substantially underpowered female ASD subgroup (n = 12), the predictor-to-mediator component (ABC → BASO) was estimated in the pooled sample. These analyses were strictly exploratory and cross-sectional; no causal ordering was implied, and the findings in girls were hypothesis-generating only. ABC, Autism Behavior Checklist; BASO, absolute basophil count; CARS, Childhood Autism Rating Scale.

Disclaimer/Publisher's Note: The statements, opinions and data contained in all publications are solely those of the individual author(s) and contributor(s) and not of MDPI and/or the editor(s). MDPI and/or the editor(s) disclaim responsibility for any injury to people or property resulting from any ideas, methods, instructions or products referred to in the content.

https://doi.org/10.3390/biomedicines14061237
